# Editorial: Methods in alloimmunity and transplantation: 2023

**DOI:** 10.3389/fimmu.2024.1516554

**Published:** 2024-11-11

**Authors:** Guido Moll, Andreas Beilhack

**Affiliations:** ^1^ BIH Center for Regenerative Therapies (BCRT); ^2^ Berlin-Brandenburg School for Regenerative Therapies (BSRT); ^3^ Julius Wolff Institute (JWI) for Musculoskeletal Research; ^4^ Department of Nephrology and Internal Intensive Care Medicine, all three part of Charité Universitätsmedizin Berlin, corporate member of Freie Universität Berlin, Humboldt-Universität zu Berlin, and Berlin Institute of Health (BIH), Berlin, Germany; ^5^ Experimental Stem Cell Transplantation Group, Departments of Internal Medicine II and Department of Pediatrics, University Hospital Würzburg, Center of Experimental Molecular Medicine, Würzburg, Germany

**Keywords:** transplantation, alloimmunity, rejection, inflammation, cell therapy, immunosuppression, immunomodulation, methods/technology

## Introduction

**Figure 1:**
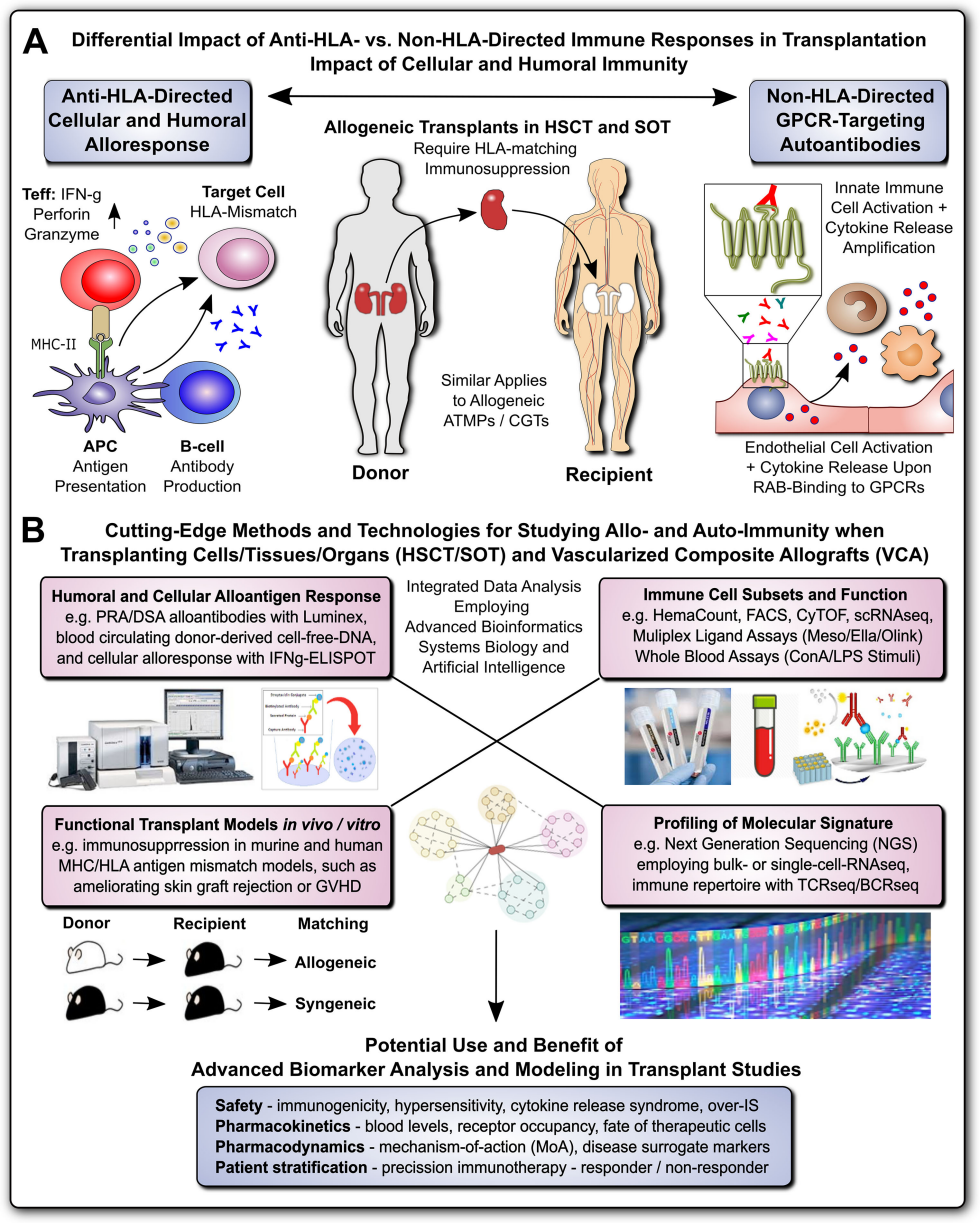
Methods for Studying Allo- and Auto-Immunity in Transplantation. **(A)** Differential Impact of Anti-HLA- and Non-HLA-directed Immune Responses in Transplantation: Allogeneic transplants in HSCT and SOT typically require HLA-matching and immunosuppression to prevent allograft rejection through anti-HLA-directed alloantigen-specific immune responses (e.g. T and B cell and alloantibody mediated), with a minor but significant contribution from non-HLA-directed auto-antigen-specific autoantibodies (e.g. GPCR-directed regulatory autoantibodies, RABs) (1, 7, 9). **(B)** Cutting-Edge Methods and Technologies for Studying Allo- and Auto-Immunity when Transplanting Cells/Tissues/Organs and Vascularized Composite Allografts (VACs): entailing at least four major important categories, such as detailed studies of: 1) Humoral and Cellular Alloantigen Responses, including monitoring of PRA/DSA with Luminex, blood circulating dd-cfDNA, and detection of cellular alloresponses with ELISpot typically IFNg-specific; 2) Functional Transplant Models *in vitro/in vivo*, including the study of novel immunosuppressive drugs and drug regimens in murine and human tissue MHC/HLA antigenic mismatch models, such as ameliorating allogeneic skin-graft rejection in mice or ameliorating GVHD in the HSCT setting; 3) Immune Cell Subsets and Function, including the use of hematology counters for absolute and relative cell quantification in whole blood, and targeted multiparametric analysis with flow cytometry/FACS and CyTOF with pre-defined panels, or broad-scale scRNAseq analysis for unbiased analysis of highly diverse cellular subsets, but also various multi-ligand-plex systems, (e.g. Mesoscale, Ella, and Olink) with different levels of sensitivity for specific ligands, and in addition whole blood assays (e.g. employing LPS or ConA stimulation for differential readout of cell type specific immune responses); and 4) Global Profiling of Molecular Signatures, including NGS-based analysis of bulk transcriptome with conventional RNAseq technology or at single-cell level with scRNAseq, and immune cell repertoire with TCRseq and BCRseq. In particular the integrated analysis of data from different analysis/modeling/readout platforms with advanced bioinformatics, including systems biology and artificial intelligence is of interest for optimal data interpretation and identification of suitable biomarkers. The potential use and benefit of advanced biomarker analysis and modeling in transplant studies entails multiple aspects, including: 1) Safety Assessment: such as immunogenicity, hypersensitivity, cytokine release syndrome, or over-immunosuppression (IS); 2) Pharmacokinetics: such as blood levels and receptor occupancy of specific ligands, or the fate of therapeutic cells; 3) Pharmacodynamics: such as studies on the mechanisms-of-action (MoA) and disease-specific surrogate markers; and 4) Patient Stratification: enabling precision immunotherapy by better understanding and distinguishing or restratifying responder and non-responder patients in advanced clinical trials. APC, antigen-presenting cell; ATMP, advanced therapy medicinal product; CGT, cell and gene therapy; MHC, major histocompatibility complex; HLA, human leukocyte antigen; HSCT, hematopoietic stem cell transplantation; SOT, solid organ transplantation; Teff and Treg, effector and regulatory T cells; GPCR, G-protein coupled receptor; RAB, regulatory autoantibodies of non-HLA type that are e.g. GPCR-directed, as distinguished from anti-HLA-directed panel-reactive alloantibodies (PRA) and donor-specific alloantibodies (DSA); dd-cfDNA, blood circulating donor-derived cell-free DNA; ELISpot-IFNg-specific, enzyme linked immune spot assay specific for release of interferon-gamma from activated T-cells; LPS, lipopolysaccharide pyrogen; ConA, concanavalin A mitogenic stimulus for T-cells; NGS, next-generation sequencing; TCRseq and BCRseq, T- and B-cell receptor sequencing, respectively.

This Research Topic is part of the “Methods in Immunology” series, which highlights cutting-edge techniques and methods used to investigate fundamental questions in immunology research, with a focus on Alloimmunity and Transplantation (**Figure 1**) (1, 2). Alloimmunity is the immune response to alloantigens – immunogenic molecules from members of the same species, including blood group antigens and the highly polymorphic antigens of the major histocompatibility complex/human leukocyte antigens (MHC/HLA) (**Figure 1A**) (3–5). Alloantigens can be classified as major and minor mismatch antigens, which is distinguished from xenoantigens/xenoreactivity against different species, and autoantigens/autoimmunity against self-antigens (1, 3–8). Alloantigens can trigger the formation of alloantibodies through alloantigen-primed B-cells and mature plasma cells (e.g. panel-reactive vs. donor-specific antibodies, PRA vs. DSA, respectively), as part of the humoral immune response, and the activation of effector T-lymphocytes, as part of the cellular immune response, with further amplification through secondary immune cell activation and infiltration (1, 3–8). Both, humoral and cellular alloimmune responses can lead to acute and chronic graft rejection in solid organ transplantation (SOT), and graft failure or graft-versus-host disease (GVHD) in hematopoietic (stem) cell transplantation (HCT/HSCT) (9–12). The most common SOT modalities include transplantation of kidneys (KTx), liver (LiTx), lungs (LuTx), heart (HTx), and vascularized composite allografts (VCA, e.g. hand, and face Tx) (1, 2, 13, 14). Successful allo-Tx requires precise “tissue matching” and optimal “IS protocols”, to reduce the risk of immune rejection and to minimize IS toxicity (15). Current advancements in alloimmunity and transplantation (**Figure 1B**) include next generation sequencing (NGS) for transcriptome analysis at both bulk or single cell levels (RNAseq and scRNAseq) (16, 17), T- and B-cell receptor repertoire sequencing (TCRseq and BCRseq) (18–20), NGS analysis of donor-derived cell-free DNA (dd-cfDNA) (21–24), sophisticated *in vitro* and *vivo* models to study transplant rejection (12, 25), but also novel concepts of transplantation (Tx), immunosuppression (IS), and patient care (15), including advanced therapy medicinal products and cell and gene therapies (ATMPs and CGTs) (1, 11, 15, 26–31). Adjunct technologies include machine perfusion of donor organs, novel renal replacement therapies (RRTs), but also the exponentially increasing use of advanced bioinformatics, systems biology, and artificial intelligence, for optimal analysis and interpretation of increasingly complex/large data sets (1, 31–37).

## Clinical application of immune repertoire sequencing in SOT

Wong et al. from the University of British Columbia and Mc Gill University in Canada, reviewed the clinical use of TCRseq and BCRseq to monitor dynamic changes in donor-reactive clonal cell populations following Tx (20), which may enable therapy adjustments to prevent rejection, reduce excessive IS, and indicate the development of tolerance. The authors reviewed 37 articles - 16 on KTx (43%) and 21 on other types (57%) and they concluded that immune repertoire sequencing is a valuable emerging tool for pre- and post-Tx monitoring.

## cfDNA quantification and qualification at the first month post lung transplant

Pedini et al. from Marseille in France conducted a prospective single-center study on 62 LuTx recipients to assess the relevance of dd-cfDNA for detecting acute and chronic rejection, or infection one month post LuTx (21–23). Total cfDNA was quantified with fluorimetry and digital PCR, cfDNA fragment size with BIABooster (Adelis), and dd-cfDNA with NGS (AlloSeq). While total cfDNA levels did not correlate with patient outcomes, higher dd-cfDNA were linked to graft injuries at d30 after LuTx (P=0.0004). A threshold of 1,72% dd-cfDNA effectively identified patients with healthy grafts, while higher levels of small dd-cfDNA indicated chronic injection or infection with 100% specificity.

## Podocytes as glomerular sentinels at the crossroads of innate and adaptive immunity

Burke et al. from the Miami Transplant Institute in Florida reviewed the role of podocytes in in focal segmental glomerulosclerosis (FSGS), a common glomerular disorder that manifests as nephrotic syndrome after KTx. They focused on podocytes as targets of circulating factors which promote recurrence of proteinuria following KTx. They discussed the potential of pre-/post-reperfusion biopsies and podocyte *in vitro* assays to develop new treatments for FSGS.

## Impact of deceased-donor characteristics on early graft function in KTx donor pairs

Mahler et al. from several Eurotransplant centers in Germany analyzed the outcomes from 328 cadaveric KTx recipients using 164 paired donor kidneys. They aimed to distinguish donor related risks from recipient and procedural variables, e.g. (a)symmetry of partner graft function, defined as early graft loss or impaired graft function (eGFR <30 ml/min) 3 months post KTx. They found that while donor factors impact early graft outcomes, they may play a limited role in long-term graft survival once the kidney graft has been accepted.

## Predicting BKV infection post KTx

Bae et al. from the Catholic University of Korea investigated whether pre-KTx polyomavirus (BKV) serostatus and BK-specific cell mediated immunity (IFNg-ELISPOT against five BK viral antigens, LT, St, VP1, VP2, and VP3) could predict post-KTx BKV infection by evaluating 93 donor-recipient pairs who underwent KTx vs. 44 healthy controls. A combination of elevated donor BKV-IgG, low recipient BKV-IgG, and low BKV ELISPOT accurately predicted BKV infections in KTx recipients, helping clinicians to intervene earlier.

## Autoantibodies from patients with KTx allograft vasculopathy promote inflammation

Moll et al. from Charité Berlin discovered that non-HLA-directed, protease activated receptor 1 (PAR1)-/G-protein coupled receptor (GPCR)-targeting regulatory autoantibodies (RABs) from KTx patients with transplant vasculopathy, but not IgG from KTx patients without vasculopathy or healthy controls, can exert immune stimulatory effects, triggering intracellular, and extracellular signaling in human microvascular endothelial cells and monocytic cells that may contribute to vasculopathy and graft failure, irrespective of alloantigen-directed responses.

## Expanded hemodialysis ameliorates uremia-induced endothelial dysfunction

Zhao et al. from Charité Berlin found that expanded hemodialysis (HDx) with medium-cutoff (MCO) membranes can reduce endothelial dysfunction caused by uremia in HD patients. In turn, HDx therapy preserved the vasculoprotective Krüppel-like factor 2 (KLF2), which counteracts inflammation and promotes vascular health.

## Better outcomes for HSCT recipients treated in home care versus hospital isolation

Ringdén et al. from Karolinska Institutet in Stockholm, Sweden, reviewed their >20-year “Karolinska Experience” providing home care to HSCT patients starting in 1998. Analyzing 252 allo-HSCT patient outcomes they found that home care is safe, reduces the risk of developing acute GVHD, lowers transplant-related mortality, improves survival, and decreases proinflammatory cytokine levels compared to hospital-treated controls.

## Autoimmune encephalitis, neurological symptoms, and neuronal antibody in HSCT

Zhang et al. from Tongji Medical College in Wuhan, China, reported a case of neuronal surface antibody syndrome (NSAS)-related autoimmune neurological disorder, with presentation of autoimmune encephalitis (AE), in a 7-year-old girl following HSCT, diagnosed with anti-metabotropic glutamate receptor-5 (mGluR5) autoimmunity, a less common form of NSAS-related autoimmunity. Treatment with IVIG and methylprednisolone, followed by oral prednisone tablets, and levetiracetam as antiepileptic therapy led to significant improvement.

## *Ex vivo* modeling of intestinal GVHD with a novel T-cell-organoid coculture system

Matthe et al. from the University Hospital Erlangen and University of Erlangen-Nuremberg in Germany developed a novel T-cell-organoid (co)culture system to study lympho-epithelial interactions in intestinal GvHD, which provides a valuable *ex vivo* platform for screening new therapeutic strategies on cellular and molecular level.

## Novel preclinical mouse model for cGVDH

Verlaat et al. from Charité Berlin report the development of two murine cGvHD models, which display high long-term morbidity, but low mortality, and depict heterogeneous clinical manifestations seen of cGVDH pathophysiology seen in patients.
